# Failure of Pavlik Harness Treatment in Infants Under 6 Months Old with Dislocated Hips: Short- and Intermediate-Term Results of Subsequent Treatment Modalities

**DOI:** 10.1007/s43465-024-01162-y

**Published:** 2024-06-07

**Authors:** Ahmet Imerci, Mihir M. Thacker, James Richard Bowen

**Affiliations:** 1 Department of Orthopaedic Surgery, Nemours Children’s Health, Delaware, 1600 Rockland Rd., Wilmington, DE 19803 USA; 2 Department of Orthopaedics and Traumatology, Faculty of Medicine, Mugla Sitki Kocman University, Mugla, Turkey

**Keywords:** Pavlik harness, Irreducible, Failure, Dislocation, Closed reduction, Complication, Open reduction

## Abstract

**Objective:**

This study aimed to determine the short- and medium-term outcomes of hip dislocation in infants who failed Pavlik harness therapy and were subsequently treated with brace, closed reduction (CR) or open reduction (OR) before 6 months of age.

**Methods:**

Fifty infants (66 hip dislocations) who failed Pavlik harness therapy between 2000 and 2018 and were treated with a rigid abduction brace or undergoing a CR or OR/cast were evaluated. All demographic data obtained from the medical system, developments and complications during the follow-up and treatment process were recorded and evaluated.

**Results:**

Fifty infants (66 hips) with dislocated hips failed Pavlik harness therapy. Of these, 9 infants (12 hips) underwent rigid abduction splint therapy: 9 hips were successful, 2 hips had CR and 1 had OR. Thirty-eight infants (51 hips) had index CR, of which 3 (3 hips) failed and had OR. Radiographs of 49 hips (44 patients) were normal at the final evaluation. Pavlik harness therapy starting after 3 weeks (*P* = 0.028) and unilateral dislocations (*P* = 0.028) increased the risk of needing operating room. There was an association between OR and avascular necrosis (*P* = 0.025), but not between OR and other complications—dysplasia and re-dislocation/subluxation (*P* = 0.257 and *P* = 0.508, respectively).

**Conclusion:**

Closed treatment of hip dislocation is possible in most babies who fail Pavlik treatment. Babies who are started on Pavlik therapy after 3 weeks of age may be at increased risk of needing an operating room.

**Level of Evidence:**

IV.

## Introduction

The goal of treatment for developmental dysplasia of the hip (DDH) is to anatomically reduce the hip joint as soon as possible to maintain normal development of the acetabulum and proximal femur. This early concentric reduction helps reduce the risk of acetabular or femoral dysplasia and gives the patient the best chance of a functional hip joint for life [1–3]. The later the diagnosis is made, the higher the complexity of interventions and the higher the risk of complications and the lower the chance of success [2–5].

Avascular necrosis (AVN) after DDH treatment is the most problematic complication in the long term. This complication can cause proximal femoral growth impairment and degenerative joint disease. AVN is a treatment-related complication [3, 6, 7]. Higher rates of AVN rates are reported in the literature after unsuccessful Pavlik harness treatment, in those who applied Pavlik harness after 6 weeks, in fixed dislocations, and in bilateral cases [3–7].

Failure of the Pavlik harness typically requires intervention in the form of CR or open reduction (OR) to achieve concentric reduction of the hip [1]. If the hip cannot be reduced with closed treatment in DDH, surgical treatment is indicated [8, 9]. During the follow-up of patients, additional corrective surgeries may be required for complications such as re-dislocation and residual acetabular dysplasia [4, 5].

The literature highlights the incidence of failure of Pavlik harness therapy for DDH, the variability in diagnosis and treatment outcomes in these failed hips, the lack of standardized management protocols, and the need for stronger supporting evidence [8, 9]. Failure of Pavlik harness therapy is observed especially in dislocated hips at rest [10, 11]. Therefore, we included only dislocated hips confirmed by dynamic ultrasonography in this study.

The aim of this study was to evaluate consecutive cases of hip dislocation (confirmed by dynamic ultrasound) infants at our institution who failed Pavlik harness therapy between 2000 and 2018, and to determine the short- and medium-term outcomes after treatment with subsequent support, CR, or OR.

## Methods

Our hospital's institutional review board approved this retrospective study (2019/1445214-Nemours Office of Human Subjects Protection). Informed consent forms were obtained from all patients participating in the study. A total of 124 (170 hips) consecutive infants with hip dislocations treated with Pavlik harnesses between January 2000 and December 2018 were evaluated. Demographic data were obtained from the patient medical records (Epic Systems, Verona, WI, USA). Patients diagnosed with hip dislocation by dynamic ultrasonography less than 6 months old, patients who underwent Pavlik harness therapy, and patients with adequate clinical and imaging follow-up for at least 2 years after the last intervention were included in this study. We excluded all syndromic dislocated hips that developed secondary to arthrogryposis, myelodysplasia, or other neuromuscular or syndromic abnormalities. Patients who were treated in other facilities before coming to our institution were also excluded from the study.

All Ortolani and Barlow examinations were performed by experienced pediatric orthopedic surgeons. Dynamic hip ultrasound was performed by pediatric radiologists using real-time sonographic equipment with 7.5-MHz linear array transducers using the technique described by Harcke and Grissom [12].

Pavlik harness treatment was started in the outpatient clinic immediately after the diagnosis of dislocation was made in all babies by clinical and ultrasonography. During the Pavlik harness treatment, the infants were followed both clinically and ultrasonographically. Pavlik harness therapy failure was defined as failure of progressive hip reduction exceeding 3 weeks after initiation of Pavlik therapy. The use of the Pavlik harness was discontinued and alternative rhino abduction braces or surgical interventions were applied according to the surgeon's preference. Ultrasound examination was performed on the reduced hips at (approximately) 3-week intervals and Pavlik harness was continued until sonographic stability was demonstrated on dynamic ultrasonography and the acetabulum returned to normal on ultrasonography.

Some patients were placed in a rigid abduction brace at the discretion of the treating surgeon. All other children were taken to the operating room for surgical reduction of the hip, either CR or OR. For hips where the Pavlik harness failed, we used an algorithmic approach to surgically reduction the hip. According to the hip arthrogram findings, all patients underwent CR with adductor tenotomy under anesthesia. If the reduction was considered acceptable and stable (according to the “safe zone” defined by Ramsey et al. [13] a spica cast was applied within the safe zone. Indications for open reduction included non-concentric, narrow safe zone. If there was (<20°) abduction or if excessive (>60°) abduction was required to keep the hip in reduction, open reduction was performed (in all cases) using an anterior approach in the same operating setting.

In the postoperative period, the hips were immobilized with a hip spica cast for at least 6 weeks. Hip position and development were then monitored using pelvic radiography every 4–6 months for the first 12 months and annually thereafter. Residual acetabular dysplasia was based on the acetabular index for age [14]. The diagnosis of AVN was made according to Salter's criteria [15]. The severity of AVN of the femoral head was assessed and classified as described by Kalamchi and MacEwen, and only grades 2 to 4 AVN were analyzed as significant [16].

Comparisons between index CR and index OR treatment were made to identify risk factors for patients requiring OR and CR. We evaluated the rates of residual dysplasia or AVN associated with different treatment modalities. All procedures performed for these complications were recorded.

### Statistical Analysis

Parametric and non-parametric analyzes were performed. Descriptive and frequency statistics were used to describe the population with mean and standard deviation. Statistical analysis was performed using SPSS v.25 (IBM, Armonk, NY, USA). Statistical comparisons between patients undergoing OR and CR were made using an appropriately unpaired t-test, Chi-square test, or Fisher's exact test. The statistical significance of the risk factors was evaluated at the 0.05 level.

## Results

**Fig. 1:**
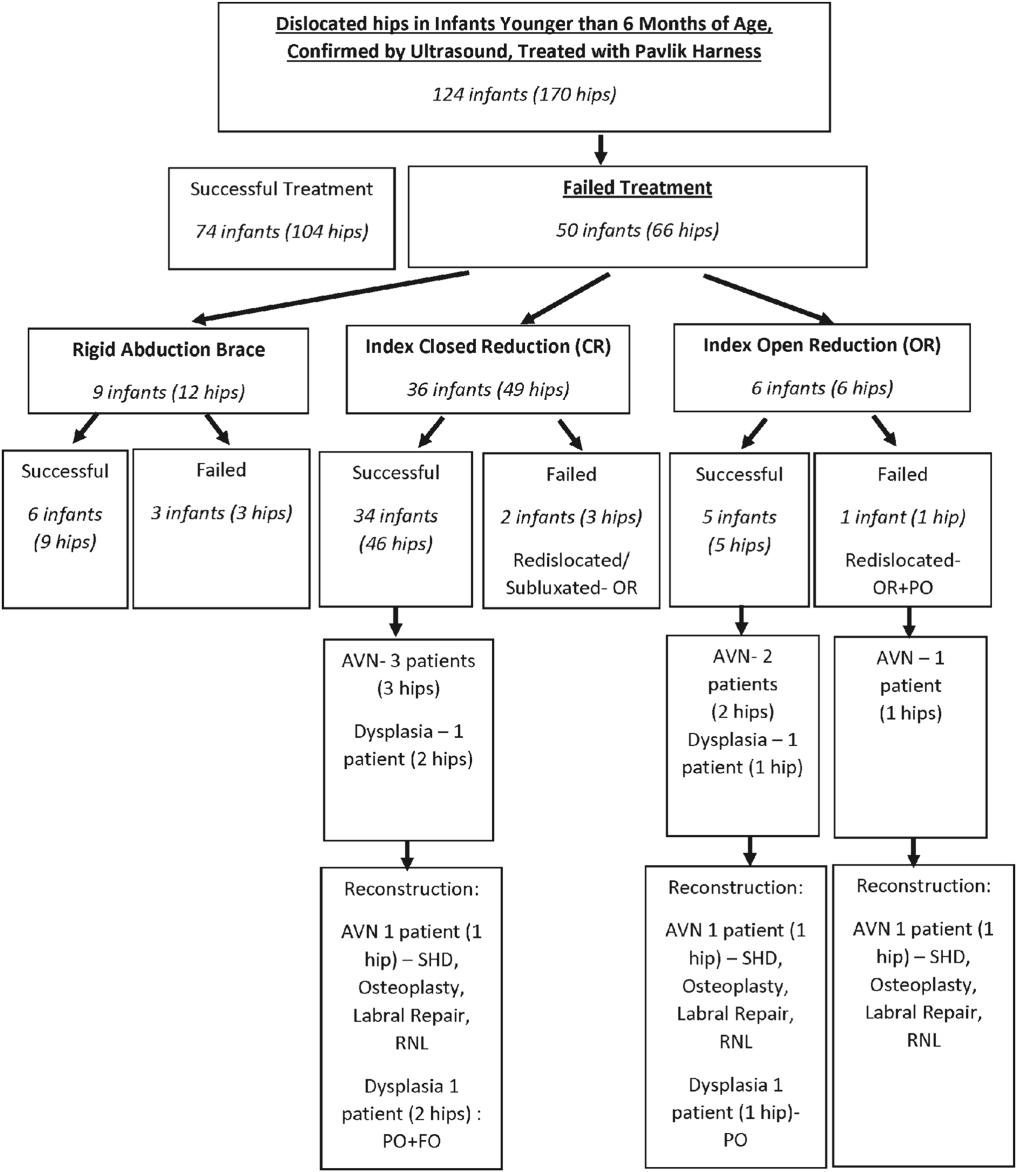
Treatment flowchart of patients with dislocated and irreducible hips with Pavlik harness. AVN indicates avascular necrosis; FO, femoral osteotomy; PO, pelvic osteotomy; RNL, relative femoral neck lengthening; SHD, surgical hip dislocation

One hundred and twenty-four (170 hip) consecutive infants with hip dislocation on both clinical and dynamic ultrasound evaluation were initially treated with a Pavlik harness and followed for at least 2 year over an 18-year period. Of these, 50 infants (66 hips, 38.8%) failed in Pavlik harness therapy. Figure 1 shows details of patient treatment modalities. The distribution of demographic characteristics of patients requiring intervention is detailed in Table 1. Twenty-three hips were reducible, but 43 were not, and intervention consisting of a rigid abduction brace, CR or OR was performed.

**Table 1:** Distribution of demographic characteristics of dislocated hips that failed Pavlik harness treatment and were subsequently treated by closed reduction or open reduction before 6 months of age

Characteristics	Patients analyzed		
	Index closed reduction	Index open reduction	Total *N*
No. of patients [*N* (%)]	38 (86.4)	6 (13.6)	44
No. of hips [*N* (%)]	51 (89.5)	6 (10.5)	57
Sex, male/female	6/32	0/6	6/38
Days of age at onset of Pavlik harness [days (range)]	24.66 ± 24.71 (3–110)	16.33 ± 12.20 (5–34)	22.68 ± 23.41 (3–110)
Average days of duration of use of harness [days (range)]	45.47 ± 37.13 (13–168)	51.17 ± 33.28 (15–104)	46.71 ± 37.58 (13–168)
Average age of operation [age days (range)]	122.26 ± 72.36 (38–381)	336.2 ± 349.14 (25–823)	147.77 ± 150.27 (25–823)
Average years of duration of follow-up, [duration y (range)]	6.11 ± 4.56 (0.6–16)	7.96 ± 6.78 (2–18)	6.72 ± 4.85 (0.6–18)
Fetal presentation [*N* (%)]			
Breech	14 (36.8)	4 (66.7)	18
Cephalic	13 (34.2)	2 (33.3)	15
Unknown	11 (28.9)	0 (0)	11
Delivery method [*N* (%]			
Cesarean	14 (36.8)	4 (66.7)	18
Vaginal	22 (57.9)	2 (33.3)	24
Unknown	2 (5.3)	0 (0)	2
Family history [*N* (%)]			
No	28 (73.7)	5 (83.3)	33
Yes	10 (26.3)	1 (16.7)	11
Multigravida [*N* (%)]			
No	23 (60.5)	3 (50.0)	26
Yes	13 (34.2)	2 (33.3)	15
Unknown	2 (5.3)	1 (16.7)	3

Nine infants (12 hips) were treated with rigid abduction braces. The abduction orthosis was successful in stabilizing 9 hips (6 patients); however, in 3 hips (3 patients), the abduction orthosis failed to stabilize/dislocate; Operative CR in 2 hips and operative OR in 1 hip were performed. Fifty-seven hips in the remaining 41 patients were successfully treated with either CR (48 hips) or OR (9 hips) (Table 2). Thirty-eight infants (51 hips) had index CR and subsequent Spica cast. Three hips failed CR and OR was performed via anterior approach and subsequent Spica casting (Table 2).

**Table 2:** Reduction technique after Pavlik harness failure in children with dislocated hips

Treatment methods	Hips *N* (%)
Index closed reduction	51 (89.5)
Successful closed reduction	48 (94.1)
Failed closed reduction	3 (5.9)
Index open reduction	6 (10.5)
Total open reduction	9 (15.8)

**Fig. 2:**
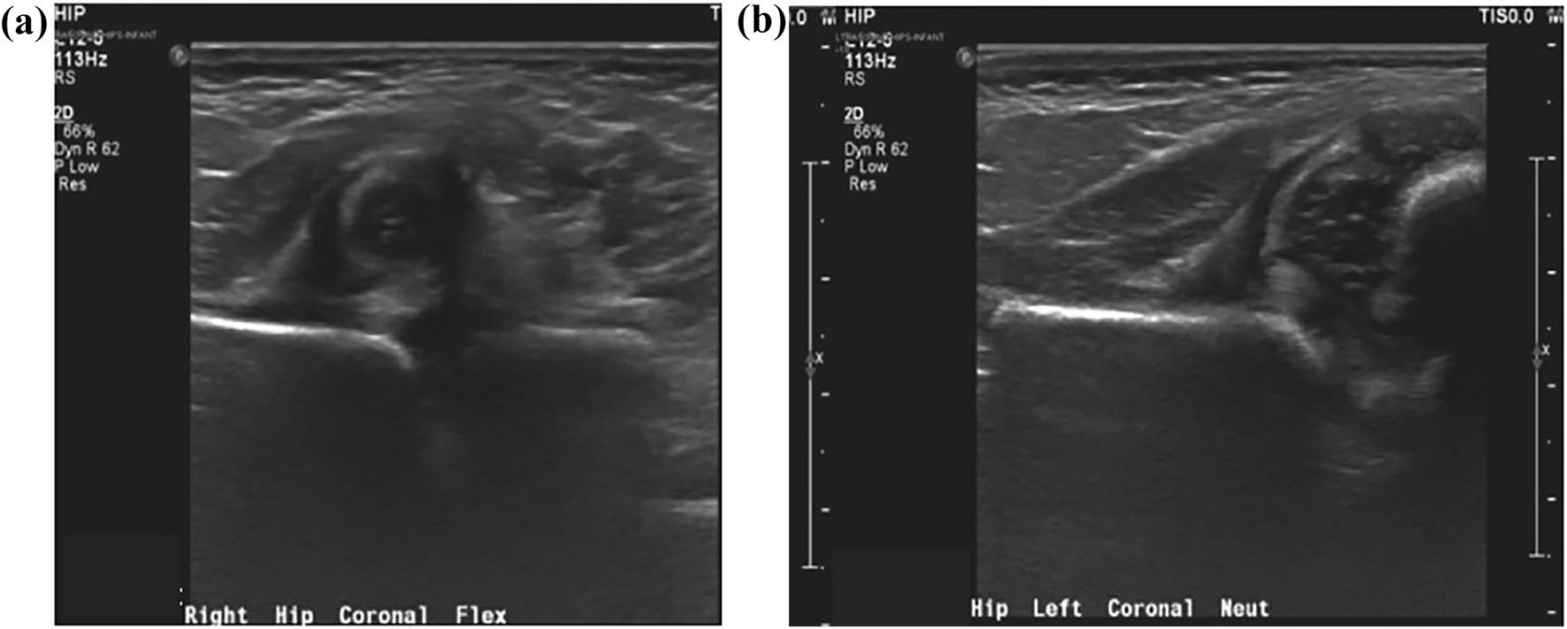
Coronal right hip (**a**) and left hip (**b**) ultrasound images of an 8-week-old girl with bilaterally frankly dislocated hips. This girl failed the 3-week Pavlik harness treatment. At 12 weeks of age, closed reduction was successful with adductor tenotomy and arthrography

**Fig. 3:**
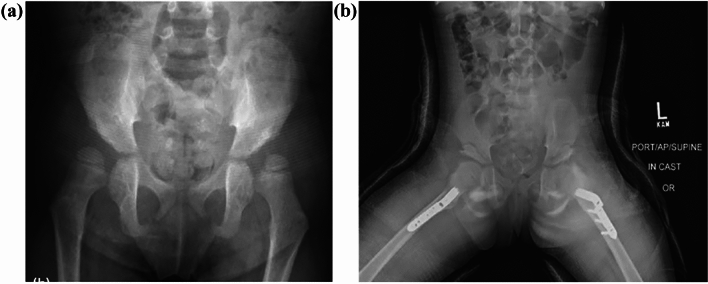
In the 2-year follow-up of the same child, bilateral acetabular dysplasia (**a**) is seen on anteroposterior radiograph of the pelvis. **b** Immediate postoperative radiograph showing bilateral Dega osteotomy and varus derotation osteotomy

During the 6.72 ± 4.85 years follow-up of these patients, at least one complication developed in 12 hips of 10 patients, and 10 hips required additional surgical procedures (OR and/or reconstruction with femoral and/or pelvic osteotomy) (Figs. 2, 3). Complications in the CR and OR groups are detailed in Table 3.

**Table 3:** Distribution of complications

Complications *N* (%)	Hips analyzed			*P* value
	Index closed reduction (*N* = 51)	Index open reduction (*N* = 6)	Total index intervention (*N* = 57)	
Avascular necrosis types 2 and 3	3 (5.9)	3 (50.0)	6 (10.5)	0.025*
Dysplasia^a^	2 (3.9)	1 (16.7)	5 (8.7)	0.257
Redislocation/subluxation	3 (6)	1 (16.7	5 (8.7)	0.508
Additional surgery–open reduction and/or reconstruction and/or pelvic osteotomy	6 (11.8)	4 (66.7)	10 (17.5)	0.007*

*Indicates statistical significance; ^a^defined by acetabular index measurement

The incidence of AVN after failed Pavlik harness therapy was 10.5% (6/57); 1.0% (1/104) after successful Pavlik harness therapy. In statistical evaluation, starting Pavlik harness treatment after 3 weeks increased the OR requirement (*P* = 0.028). Unilateral dislocation was also a risk factor for OR (*P* = 0.028) (Table 4). While an association was noted between OR and AVN (*P* = 0.025), we did not find any association between OR and other complications, including dysplasia and re-dislocation/subluxation (*P* = 0.257 and 0.508, respectively). There was a statistically significant correlation between OR due to existing complications and the need for additional surgery (*P* = 0.007) (Table 3). There was no significant relationship between AVN and unilaterality and age at initiation of Pavlik harness therapy (*P* = 0.624 and *P* = 0.376, respectively).

**Table 4:** Statistical analysis of patient factors affecting choice of interventions

Patient factors	Hip analysis		
	Index closed reduction (*N* = 51)	Index open reduction (*N* = 6)	*P* value
Age in weeks at onset of Pavlik harness treatment [*N* (%)]			
≤3 weeks	35 (68.6)	3 (50.0)	0.028*
3 weeks	16 (31.4)	3 (50.0)	
Average days of duration of use of harness [duration days (range)]	45.47 ± 37.13 (13–168)	51.17 ± 33.28 (15–104)	0.697
Bilateral [*N* (%)]	13 (34.2)	0 (0)	0.028*
Unilateral [*N* (%)]	25 (65.8)	6 (100.0)	
Side [*N* (%)]			
Right	21 (41.2)	2 (33.3)	0.412
Left	30 (58.8)	4 (66.7)	
Sex [*N* (%)]			
Female	32 (84.2)	6 (100.0)	0.391
Male	6 (15.8)	0 (0)	
Multigravida [*N* (%)]			
No	23 (60.5)	3 (50.0)	0.365
Yes	13 (34.2)	2 (33.3)	
Unknown	2 (5.3)	1 (16.7)	

^*^Indicates statistical significance

## Discussion

This study comprised a large cohort of patients investigating initial Pavlik harness therapy in 124 consecutive infants (170 hips) with significant hip dislocation confirmed by sonographic examination at diagnosis. Our failure rate of 33.6% is among the lowest reported in the literature for the most severe form of DDH [10, 17–19]. The type of hip dislocation may also have affected this rate. In our series, 57 hip interventions were required in 44 patients.

### Rigid abduction support in patients failing Pavlik harness therapy

Hedequist et al. [20] reported that 14 hips (13 Ortolani positive and 1 Barlow positive) were treated with rigid abduction orthosis after unsuccessful Pavlik harness therapy. Eleven of 14 hips were successfully treated, and the remaining three hips (2 patients) had successful CR. On the contrary, Abraham et al. [8] pavlik reported that all seven patients with dislocated/dislocated hips who failed Pavlik harness therapy also failed in the rigid abduction brace; of these patients, 4 had successful CR and 3 required OR. Novias et al. [11] Pavlik harness failed in 21 (26.9%) of 78 Ortolani hips, and 9 (42.9%) of them were treated with a rigid abduction brace, 4 of them CR and 8 of them OR. Similarly, Gornitzky et al. [21] reported a multicenter study of 38 patients (49 hips) who failed Pavlik harness therapy. Of these, 44.9% (22/49) were successfully treated with a rigid abduction brace. Similarly, the success rate of the abduction brace in our study was quite high with 75.0% (9/12). It failed in three of four reduced hips and one of eight reduced hips. However, a rigid abduction brace was not applied to all patients who failed Pavlik harness therapy.

### Subsequent Treatment of Patients Failing Pavlik Harness Therapy

Arneill et al. [22] reported their cohort of 67 hips that had recently failed the Pavlik treatment. They were able to perform CR in 46 (68.7%) of 67 hips, of which 11 hips were reduced, giving them a successful CR percentage of 52.2% (35/67 hips). Aarvold et al. [10] reported that Pavlik harness therapy failed in 19 (39.6%) of 48 dislocated, irreducible hips that were subsequently successfully treated with CR (7 hips) or OR (12 hips). Two hips developed AVN—1 managed with CR and 1 managed with OR. In a similar cohort that included 166 hips of 154 patients, Talati et al. [9] reported nonoperative treatment failure in 81 hips of 68 patients. Overall, 34 (42.0%) of these hips required an operating room. Only Graf III and IV classification has been identified as an important determinant for OR need. These authors found that age and duration of orthotic treatment did not affect the choice of reduction technique [9]. In another study, the Pavlik harness failure rate was found to be 14.0% (30/215) for all DDH types, and the OR requirement was reported to be 26.7% [11]. In a study involving Ortolani-positive neonates, the authors found that the Pavlik harness failed to reduce and stabilize the hip 25.8% (8/31) of which 2 were treated with CR and 6 required OR. They also reported that AVN developed in the femoral head in 2 of 6 cases with OR. Gornitzky et al. [21]. of the failed Pavlik harness treatment cohort, 77.6% reported successful treatment using closed methods (44.9% rigid abduction brace and 32.7% CR), and 20.4% went to the operating room (Table 5). Senaran et al. [7] stated that only 2 (5.7%) of the 35 hips Pavlik failed needed surgery. Of the 44 patients in our cohort who required operating room, 38 (86.3%) had successful reduction with CR and 6 (13.7%) with OR. We attribute this success rate to the early age of reduction, but this must be carefully balanced with concerns about the effect of anesthesia on the developing brain.

**Table 5:** Study characteristics of Pavlik harness treatment failures

Study	Hip types included in the study	Number of dislocated hips	Numbers of hips failing Pavlik harness	Dislocated hips managed by alternative first-line treatments to Pavlik harness	Additional operations	Avascular necrosis	Risk factor for OR
Aarvold et al. [10]	Irreducible hips	48 hips	21 (43.7%)	7 CR	NR	2 patients after CR	NR
				12 OR			
						2 patients after Pavlik	
Arneill et al. [22]	Dislocated hips	543 (644 hips)	67 hips	35 CR	3 pelvic	Total 26 (39%) AVN (grade 1 in 21 hips (81%), 2 in 1 hip (4%), 3 in 3 (12%), and 4 in 1 hip (4%)	NR
				32 OR	2 varus derotational femoral osteotomy		
Gornitzky et al. [21]	Dislocated hips	-	38 (49 hips)	22 (45%) brace	1 patient (2%) revision OR	NR	NR
				16 (33%) CR			
				10 (20%) OR			
				1 no further treatment			
Talathi et al. [9]	Graf III and Graf IV dislocated hips	68 (81 hips)	62 hips	28 (45%) CR	None	NR	Ortolani test (−)
				34 (55%) OR			
Novais et al. [11]	Ortolani ( +) dislocated hips	59 (78 hips)	17 (21 hips)	9 brace	None	NR	NR
				4 CR			
				8 OR			
Tiruveedhula et al. [6]	Dislocated hips	123 hips	37 hips (30%)	20 CR		5 hips AVN after CR	
				17 OR			
						5 hips AVN after OR	
Liu et al. [25]	Graf III and Graf IV dislocated hips	35 hips	25 hips	36 CR	6 pelvic osteotomy	6 hips AVN	Not using Pavlik harness
				6 OR			
Our study	Dislocated hips	124 (170 hips)	50 infants (66 hips, 38.8%)	9 (13.6%) brace	4 pelvic osteotomy	3 AVN after CR	Onset Pavlik harness treatment after 3 weeks and unilateral dislocations
					3 reconstruction	3 AVN after OR	
				48 (72.7%) CR			
				9 (13.7%) OR			

*AVN,* avascular necrosis; *CR,* closed reduction; *NR,* not or inadequately reported; *OR,* open reduction

### Avascular Necrosis After Unsuccessful Pavlik Harness Therapy

After failure of Pavlik harness therapy, the incidence of AVN in cases with success with other treatment modalities ranges from 14.0 to 33.0% in most large series [6, 7, 20, 23]. Tiruveedhula et al. [6] reported that CR was required in 20 (54.1%) of 37 hips that failed Pavlik harness therapy, and OR was present in 17 of them at 10-year follow-up. They found the overall AVN rate to be 27.0% and stated that the reduction technique did not affect it. They reported that the risk of AVN was 4 times higher than in patients who presented late after unsuccessful Pavlik harness treatment and underwent surgical treatment alone [6]. In our study, although there was no significant difference between the CR and OR groups in terms of the duration of Pavlik harness use, we found that the AVN rate was significantly higher in the OR group, but the numbers in the OR group were very low. We attribute this to the fact that these are the hips most severely affected and cannot be closed. We found that the duration of use of Pavlik harness therapy did not affect the mode of reduction (*P* = 0.697), but treatment started after 3 weeks was a risk factor for OR (*P* = 0.028). Interestingly, unilateralism was also a risk factor for OR (*P* = 0.028), although this is probably related to sample size.

Sankar et al. [4] included 78 patients younger than 12 months (87 hips), with a median age at first CR of 8 months. In 79 hips (91%), CR was initially successful, but 79 hips (8.9%) had hip redislocate and required OR. During the short-term follow-up (12–36 months), 8 out of 72 (11.1%) hips with successful CR reported acetabular and/or femoral osteotomy for residual dysplasia and 18 out of 72 AVN (25.0% hips). They found no relationship between the development of AVN and previous brace use and CR age [4]. In our study, which had a mean follow-up of 6.72 years, successful CR was achieved in 48 of 51 hips and additional surgery (femoral and/or pelvic osteotomy) was achieved in 6 hips (12.5%). On the other hand, four of six patients with OR initially underwent surgical intervention. Considering the difference in follow-up periods in this study [4], our AVN rate is relatively lower, at 10.5%.

Our data also show that dislocated hips in infants less than 6 months of age are less likely to fail CR [5.9% (3/51)] and require an operating room [15.8% (9/57)] at index surgery. Young age at CR (122.26 ± 72.36 days) in these patients likely contributed to the high success rate of CR. Infants with unilateral disease who were started on Pavlik harness therapy after 3 weeks of age were more likely to have OR. As in previous series, we did not find a significant relationship between Pavlik harness treatment time and the need for CR and OR [17, 24]. Liu et al. [25] stated that the use of the Pavlik harness in patients treated with CR/OR before 6 months significantly reduced the need for surgery, but had no effect on complications in the follow-up.

Due to the retrospective nature of this study, there are some limitations that prevent coherent decisions regarding the discontinuation of the Pavlik harness. However, Pavlik harness time is consistent with most large studies. Also, the mean follow-up is only 6.72 years, which would probably preclude us from getting a good idea of the true prevalence of AVN or proximal femoral growth disorders due to treatment factors. However, follow-up is sufficient so that we can catch more severe/clinically relevant AVN cases and assess the possibility of needing early reconstructive surgery for acetabular dysplasia. The uniformity of the cohort in terms of severity of ultrasound-documented hip dislocation and age less than 6 months at the initial evaluation prior to starting Pavlik therapy allows us to meaningfully compare the results of abduction support, CR, and OR.

## Conclusion

Based on the available data, we recommend that families of infants <6 months of age with hip dislocation/unreducible who fail Pavlik harness therapy be consulted about the high success rate and low complications of closed therapy in the medium term.
